# Human neural stem cell transplantation in ALS: initial results from a phase I trial

**DOI:** 10.1186/s12967-014-0371-2

**Published:** 2015-01-27

**Authors:** Letizia Mazzini, Maurizio Gelati, Daniela Celeste Profico, Giada Sgaravizzi, Massimo Projetti Pensi, Gianmarco Muzi, Claudia Ricciolini, Laura Rota Nodari, Sandro Carletti, Cesare Giorgi, Cristina Spera, Frondizi Domenico, Enrica Bersano, Francesco Petruzzelli, Carlo Cisari, Annamaria Maglione, Maria Francesca Sarnelli, Alessandro Stecco, Giorgia Querin, Stefano Masiero, Roberto Cantello, Daniela Ferrari, Cristina Zalfa, Elena Binda, Alberto Visioli, Domenico Trombetta, Antonio Novelli, Barbara Torres, Laura Bernardini, Alessandro Carriero, Paolo Prandi, Serena Servo, Annalisa Cerino, Valentina Cima, Alessandra Gaiani, Nicola Nasuelli, Maurilio Massara, Jonathan Glass, Gianni Sorarù, Nicholas M Boulis, Angelo L Vescovi

**Affiliations:** Department of Neurology, Eastern Piedmont University, Maggiore della Carità Hospital, Corso Mazzini n. 18-28100, Novara, Italy; Laboratorio Cellule Staminali, Cell Factory e Biobanca, Terni Hospital, via Tristano di Joannuccio 1, 05100 Terni, Italy; IRCCS Casa Sollievo della Sofferenza, viale dei Cappuccini, 71013 San Giovanni Rotondo, Foggia, Italy; Biotechnology and Bioscience Department Bicocca University, Piazza della Scienza 2, 20126 Milan, Italy; Department of Neuroscience, “Santa Maria” Hospital, Terni via Tristano di Joannuccio 1, 05100 Terni, Italy; Department of Physical Therapy, Maggiore della Carità Hospital, Corso Mazzini n. 18-28100, Novara, Italy; Department of Diagnostic and Interventional Radiology, “Eastern Piedmont” University, “Maggiore della Carità” Hospital, Corso Mazzini n. 18-28100, Novara, Italy; Department of Neuroscience, University of Padova, Via Giustiniani, 2 – 35100, Padova, Italy; Department of Neurology Emory University, 201 Dowman Dr, Atlanta, GA 30322 USA; Department of Neurosurgery Emory University, 201 Dowman Dr, Atlanta, GA 30322 USA; Fondazione Cellule Staminali di Terni, Terni Hospital, via Tristano di Joannuccio 1, 05100 Terni, Italy

**Keywords:** Phase I trial, Foetal human neural stem cells, Cell therapy, ALS, Advanced therapies

## Abstract

**Background:**

We report the initial results from a phase I clinical trial for ALS. We transplanted GMP-grade, fetal human neural stem cells from natural in utero death (hNSCs) into the anterior horns of the spinal cord to test for the safety of both cells and neurosurgical procedures in these patients. The trial was approved by the Istituto Superiore di Sanità and the competent Ethics Committees and was monitored by an external Safety Board.

**Methods:**

Six non-ambulatory patients were treated. Three of them received 3 unilateral hNSCs microinjections into the lumbar cord tract, while the remaining ones received bilateral (n = 3 + 3) microinjections. None manifested severe adverse events related to the treatment, even though nearly 5 times more cells were injected in the patients receiving bilateral implants and a much milder immune-suppression regimen was used as compared to previous trials.

**Results:**

No increase of disease progression due to the treatment was observed for up to18 months after surgery. Rather, two patients showed a transitory improvement of the subscore ambulation on the ALS-FRS-R scale (from 1 to 2). A third patient showed improvement of the MRC score for tibialis anterior, which persisted for as long as 7 months. The latter and two additional patients refused PEG and invasive ventilation and died 8 months after surgery due to the progression of respiratory failure. The autopsies confirmed that this was related to the evolution of the disease.

**Conclusions:**

We describe a safe cell therapy approach that will allow for the treatment of larger pools of patients for later-phase ALS clinical trials, while warranting good reproducibility. These can now be carried out under more standardized conditions, based on a more homogenous repertoire of clinical grade hNSCs. The use of brain tissue from natural miscarriages eliminates the ethical concerns that may arise from the use of fetal material.

**Trial registration:**

EudraCT:2009-014484-39.

## Background

Amyotrophic Lateral Sclerosis (ALS) is a devastating, incurable neurodegenerative disease that targets motor neurons (MNs) in the primary motor cortex, brainstem, and spinal cord. Cell therapy is emerging as a potential therapeutic option in ALS, although numerous scientific, technical, ethical and regulatory issues remain to be overcome. One critical issue pertains to the type of stem cells to be used in cell therapy for ALS. Donor cells must not only survive in the human central nervous system (CNS), but ought to be capable of improving the tissue pathophysiological condition, possibly by modulating local inflammatory and immune reactions and by antagonizing toxic phenomena [1,2]. Notwithstanding, the first critical requirement that stem cells must meet in clinical applications concerns their safety. At the same time, a pressing need exists for them to be easily isolated and reproducibly and stably expanded *ex vivo* [3]. Thus, both hematopoietic and mesenchymal stem cells (MSCs) have now been transplanted into the spinal cord of ALS patients, in the absence of long-term negative effects [4-6]. Others and we have previously documented the integration capacity and prospective therapeutic efficacy of human neural stem cells (hNSC) in preclinical rodent models of neurological diseases [7-10]. Of interest, we showed that, when implanted either intravenously or intrathecally, NSC ameliorate the pathophysiological and neurological traits in an experimental model of autoimmune encephalomyelitis, both in rodents [11] and non-human primates [12]. A key conclusion from these studies was that implanted NSCs would integrate and survive predominantly as astroglial cells. These would likely elicited their effects through the release of growth factors and immunomodulatory molecules [12].

The injection of candidate therapeutic cells in the proximity of the degeneration site(s) may better suit the key requirements in the neural transplantation context. In ALS, engraftment of donor cells close to the dying MNs might favour the diffusion of trophic and immunomodulatory factors to both the MNs themselves and the surrounding glial cells, thereby enhancing the likelihood of accomplishing therapeutic effects. Accordingly, a meta-analysis of 11 independent studies demonstrated that, when implanted close to the dying MNs, NSCs may slow both the onset and the progression of clinical signs and can prolong overall survival in ALS mice [13]. Furthermore, it has recently been shown that transplantation of human neural progenitor cells into the ventral cervical spinal cords of SOD1G93A rats can slow the death of phrenic motor neuron and increase activity in spared phrenic MNs [14].

A technique for the focal delivery of donor cells in the proximity of ventral MNs has recently been established using a stabilized, stereotaxic frame [15]. This system, previously standardized in animal models [16,17], has now been employed in the first FDA-approved cell-therapy trial for ALS, implanting human neural progenitor cells [18,19].

Here we expand on these studies and report the preliminary results from a first group of six ALS patients in an ongoing Phase I trial, in which *bona fide*, multipotent hNSCs were isolated and reproducibly expanded from human fetal tissue. These cells were obtained from spontaneous miscarriages [8] and implanted using a stereotaxic/surgical apparatus and injection procedures similar to those used by Riley and colleagues [17].

## Methods

The study was performed as a prospective, open pilot study. The trial was approved and monitored by the competent Italian authorities, i.e. the Istituto Superiore di Sanità (ISS), Agenzia Italiana del Farmaco (AIFA, member of the European Medicine Agency) and by the Ethics Committees of the Umbria Region, of the “Maggiore della Carità” and of the “San Antonio” Hospitals. It goes by the European Clinical Trials Database (*EudraCT*) identification number 2009-014484-39. All patients provided written, informed consent. All the recorded data for the recruited patients concerning the whole follow-up period were registered in the “*Database For Clinical Studies With Gene And Somatic Therapy*” of the ISS. The same data were also communicated to an independent Safety Monitoring Board of multidisciplinary experts, whom controlled strict compliance with the protocol and monitored possible adverse events.

### Patients

Patients were deemed eligible when they had definite or probable sporadic ALS, according to the El Escorial Revised Criteria [20]. Given the high iatrogenic risk, patients in this initial study were selected whom suffered from severe functional impairment in their lower limbs. The detailed list of inclusion and exclusion criteria is reported in Table 1. All patients received ordinary medical treatment, including riluzole.

**Table 1:** **Inclusion and exclusion criteria**

**Inclusion Criteria**	**Exclusion Criteria**
Age 20 to 75 years	Psychiatric disease or other neurological disease different from ALS
Non ambulatory (walking subscore 0–1 of the ALS-FRS-R scale)	Evidence of any concurrent illness
Evidence of progression disease in the last six months	Patients receiving corticosteroids, immunoglobulin or immunosuppressive treatment**
Absence of sleep apneas or hypopneas with blood oxygen saturation lower than 90%,*	Mental deterioration or cognitive sphere disturbance
FVC higher than 60%	Non-invasive ventilation (NIV) >6 hours daily Patients unable to understand informed consent form and study aims
Supposed good adherence to study protocol	Women who were pregnant or childbearing potential for the duration of the study.
Good acceptance and understanding of the informed consent	

*nocturnal respiratory monitoring by polysomnography.

**We excluded patients who received these treatments in the 6 months prior to the transplant.

We expected a large impact on the mass media and inferred that this kind of trial would raise significant expectations in a large population of patients. Hence, in order to deal with the likely rather large number of possible applications, a toll-free phone number and a web-based portal were set up. Thorough this, patients could apply for admission to the trial by filling out a form and by sending a standardized set of clinical information for initial screening. The neurologists of the staff reviewed these submissions and eligible patients were summoned for a visit at the recruitment centres, until the maximum number of patients allotted by the recruitment criteria was reached.

Case-reports for eligible patients were sent to the ISS, whose experts verified the appropriate adherence to the protocol and authorized the treatment. At this point the patients were informed of their recruitment into the study.

The informed consent was structured as an interview, which openly and clearly stated the experimental and preliminary nature of our clinical study and the risks associated with the procedure. The neurologist discussed each question with the patients and their relatives. This procedure was in agreement with the recommendations of the International Society for Stem Cell Research [21]. Subjects were made aware that their participation in this study was entirely voluntary and that participation or non-participation would not interfere with their ongoing clinical care. Before signing their consent, patients and close relatives were offered the possibility of meeting separately with their family physician, a neurosurgeon and a consultant neurologist, whom were in no manner involved in our trial, in order to discuss all pending issues. A psychologist closely examined the patients before and immediately after recruitment. A clinical interview and the MMPI-2 test were used to ensure that the participants fully comprehended that this was a safety trial and all of the risks associated with the procedure.

### Study sites

Patients were enrolled and evaluated at the Tertiary ALS Centres in Novara and Padova, Italy. All surgical procedures were performed at the “Santa Maria” Hospital in Terni, Italy, in close proximity to the cell factory that prepared and released the GMP-grade hNSCs upon the formal authorization granted by AIFA (protocol number aM 02/2014).

### Safety measures

The primary outcome measure was the immediate and long-term safety profile. Mortality from any cause and serious adverse effects were the primary occurrences that were assessed. Patients were closely monitored for immediate adverse events, including allergic reactions (tachycardia, fever), respiratory failure, local complications (intraparenchymal hematoma, local infection at the site of surgery), systemic complications (systemic infections), paralysis or sensory loss below the level of the injection site. Potential, delayed adverse events included intraspinal tumor formation, aberrant connections (spinal myoclonus), persistent sensory loss or paralysis not related to the progression of the disease.

The secondary outcome measures were the difference in functional outcomes measured by the ALS-FRS-R scale and forced vital capacity (FVC).

### Clinical assessment and follow-up

To estimate the rate of disease progression, patients were monitored monthly for 1 year and then every three months, until death. After enrollment, but prior to surgery, each patient was subjected to a three-month observation period. Clinical progression was assessed using the ALS-FRS-R, Ashworth Spasticity Scale and the Medical Research Council (MRC) scale of 34 muscle groups of the upper and lower limbs and FVC. At each examination a clinical psychologist also evaluated the patients. Profile of Mood State (POMS) [22] and SEIQoL-DW [23] questionnaires were provided to patients to assess the mood state and the quality of life.

### Neuroimaging

**Figure 1:**
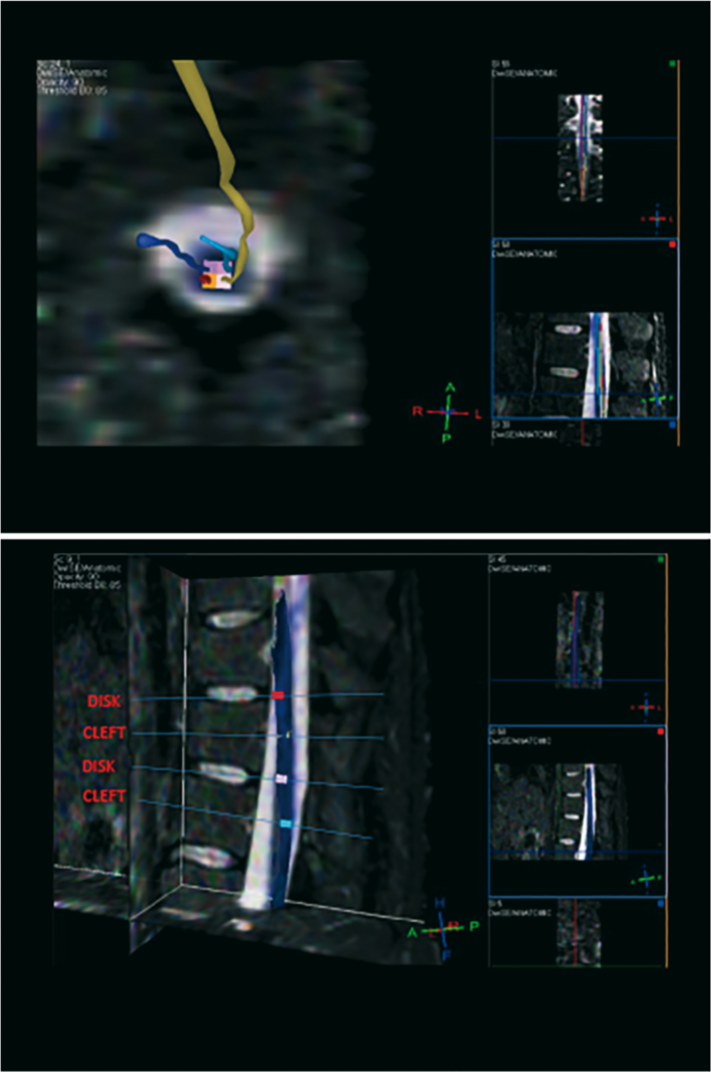
**Spinal cord MRI. Upper Panel**: Diffusion Tensor Imaging (DTI) overlaid with the STIR pulse sequence, in a MPR algorithm. For each of single point (colored box) ROIs, a correspondent fiber is evoked and reconstructed to ensure that the level examined and the ROI adopted is inside the spinal cord. **Lower Panel**: DTI post-processing by mean of a MPR algorithm, with overlaying of the STIR pulse sequence, to select the exact levels to be studied at this time of examination and in further MR follow-up scans. Disk and somatic vascular cleft are adopted to select the proper planes.

Brain and spinal cord (SC) MRIs were obtained using a 1.5-T imaging system (Achieva Intera, Philips). Spinal cord MRIs were performed pre-operatively and at 21 days and at 3, 6, 9 and 12 months after surgery. Brain MRIs were performed at the time of entry in the study and 12 months after surgery. In addition to the full conventional MRI, the spinal cord was imaged by a diffusion tensor imaging (DTI) pulse sequence in the axial plane, with 64 directions, in order to identify and quantitatively characterize the tissue. Using the fiber-tracking algorithm, we calculated the fractional anisotropy (FA) and apparent diffusion coefficient (ADC) values. We used FiberTrack Software (Philips, Best, The Netherlands) and, after a test/re-test for intra-individual variability evaluation, we measured FA and ADC at the implant site (T11-T12) and in normal tissue in the upper cervical and lower lumbar spinal cord segments, by placing single point ROIs. To ensure that the chosen ROIs were effectively inside the spinal cord and part of its architectural structure we detected and reconstructed a spinal fiber bundle at each ROI. If not evoked, we discarded that ROI changing its position (Figure 1 upper panel). To ensure that at every follow-up the same axial level would be selected, we chose the vascular cleft of the vertebral body and the midline inside the intervertebral disk between the somatic endplates as reference sites (Figure 1 lower panel).

We overlaid, inside a Multi-Planar Reformat (MPR) program, a high resolution axial T2 weighted image on the volumetric DTI data. Measurements were carried out placing two ROIs, right and left, localized in the supposed site of implantation (the grey matter of the anterior horns of the spinal cord). The mean of the two measures was considered in the analysis for the anterior part of spinal cord. Another two ROIs were drawn in correspondence to the posterior spinal bundles, making for a total of four ROIs for each level.

### Blood tests

Blood routine laboratory tests (renal function, liver function, glucose) and test for Hepatitis B or C, HIV and TBC were performed at the study entry and 7 days before surgery. Routine and haematological (full blood count and CD3+, CD3+/CD8+, CD4+/8+ counts) laboratory tests and tacrolimus dosage were performed weekly during the first post-operative month and then monthly during the 6 months of the immunosuppression period.

### Urinary bladder evaluation

Bladder ultrasound, measuring the post void residual volume, was performed at study entry and on the 2nd and 21st days after surgery and then every three months, for one year.

### Human neural stem cells

The hNSCs used in this clinical trial were produced according to good manufacturing practice protocols (GMP), as dictated by the European Medical Agency (EMA) guidelines. The tissue collection procedure, the cell factory, the production procedure and the cell validation criteria received formal approval and certification by the appropriate regulatory body, namely the Agenzia Italiana del Farmaco, protocol number aM 101/2010 (updated in 2014 after AIFA inspection to number aM 2/2014). Tissues and cells were procured and handled as described below [24].

#### Human fetal tissue

Human fetal brain tissue specimens, all derived from the forebrain, were routinely collected from fetuses at gestational ages greater than the 8th post-conceptional week. They were immediately dissected and used to generate hNSC lines under sterile conditions. Tissue procurement was approved by the ethical committee of the Institute “Casa Sollievo della Sofferenza di San Pio da Pietrelcina” and was possible exclusively upon the mother giving informed, written consent. Of note, no tissue from therapeutic or procured abortion was used in this study and all of the specimens were collected from fetuses that underwent natural, spontaneous *in utero* death, (miscarriage). Also, specimen collection and medical procedures were in full accord with the Helsinki declaration (WMA Declaration of Helsinki - Ethical Principles for Medical Research Involving Human Subjects). Dissociation of brain tissue, primary culturing and cell propagation were carried out according to the procedure described previously by Vescovi and colleagues [8]. As a whole, the results reported in this study refer to cells from a maximum of two donors, whom died at the 15th and 16th gestational week. The two different cell lines were used randomly to treat patients.

#### Primary culturing

Tissue was washed in a PBS solution (Dulbecco’s PBS 1X, PAA plus 50 ng/ml of gentamicin) and mechanically dissociated. Cells were seeded at a density of 10^4^ cells/cm^2^ as described previously [8]. Cultures were maintained in a humidified incubator at 37°C, 5% O_2_ and 5% CO_2_ and allowed to proliferate as free-floating clusters (neurospheres).

#### Cell propagation and expansion

7–10 days after the primary cell seeding the neurospheres that had formed were collected and mechanically dissociated and replated at the same initial density. This step (passaging) was routinely repeated up to 17 times before the cells were supplied to the surgery site on the day of transplantation. Throughout these passages aliquots of cells were frozen as neurospheres, which were cryopreserved in 10% DMSO (Dimethyl sulfoxide, LiStarFish) culture medium as a pharmaceutical intermediate product, with an assigned batch number. This freezing step was conducted in order to coordinate the timing of cell production with the surgery schedule. It also allowed for the establishment of an “Intermediate Product” to warrant the series of quality controls required to certify the safety, identity, potency and the pharmaceutical grade of the donor hNSCs, to satisfy the AIFA, GMP regulatory process criteria. Once the date of the surgery had been determined, the intermediate product was thawed and cells underwent additional passages and expansion (a maximum of 17 times overall, as stated above), until they were delivered to the surgery room as the “final product” (cell drug) to be transplanted.

### Cell quality control

In agreement with the AIFA certification criteria, we subjected cells to serial quality control testing throughout the whole production process (IPCs-In Process Controls). Additional control tests were performed on the Intermediate Product and Finished Product before their final release.

The “in process” controls were meant to pursue a microbial monitoring of the product (Total Microbial Count) and to evaluate cellular growth parameters (cell counting and trypan blue exclusion method) throughout all cell passages. Standard haematoxylin and eosin staining and histological evaluations were also carried out on the fetal donor tissue to confirm the neural nature of the starting material. This was in addition to the precise anatomical dissection criteria used to isolate the initial tissue. The release tests, performed at the very last passage, were meant to verify the sterility of the product, the absence of mycoplasma and a low level of endotoxin contamination (LAL test; < 1EU/ml) according to European Pharmacopeia. A critical parameter such as the “stemness” of the intermediated products and of the cells in the cell drug suspension was routinely certified by monitoring the most critical hNSCs parameters, such as self-renewal activity, differentiation capacity and growth factor dependence.

#### Self-renewal

**Figure 2:**
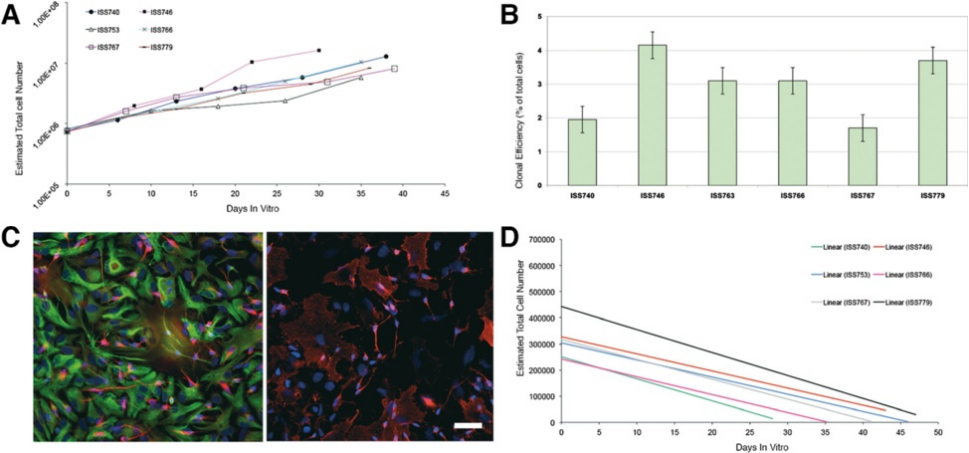
**Cell quality control. (A)** Growth kinetics of a set of hNSCs lines showing the increasing, estimated overall cell number at each passage. **(B)** Clonal efficiency assay showing the percentage of cells that retain the ability to form clonal neurospheres over the total cell number plated is reported (Replicates n = 3), bars describe standard error. **(C)** hNSCs differentiate into astrocytes (left, green, GFAP), neurons (left, red, βIII-tubulin) and oligodendrocytes (right, GalC, red); nuclei are counterstained in blue (DAPI). (Bar = 50 μm). **(D)** All of the hNSCs lines tested undergo extinction in vitro upon growth factor removal, as shown by the negative growth kinetic in which the total cell number approaches zero in a few passages.

In the culture system at hand, this could be extrapolated by determining the cells growth kinetic. In fact, in the neurosphere system, the slope of the growth curve provides an indirect index of the hNSCs’ inherent ability for self-renewal [25,26]. We determined such kinetic curve by calculating the estimated overall cell number increase at each passage, as described earlier [8] and did confirm the long-term stability of these cultures with respect to this parameter (Figure 2A).

#### Clonal efficiency assay

The analysis of the clonogenic capacity throughout passaging was used to directly confirm the stability of the self-renewal capacity of our hNSCs. This was determined by the hNSCs clonal efficiency assay, which shows the percentage of plated cells that retains the ability to form clonal neurospheres under stringent culture conditions. Briefly, neurospheres were mechanically dissociated and cells were counted, also assessing cell vitality by trypan blue exclusion. Cells were seeded at a density of 250 viable cells/cm^2^ on a Poly-L-lysine layer (Sigma-Aldrich) in a flat bottom 24-well dish and incubated at 5%O_2_ 5%CO_2_ and 37°C in DMEM/F12, in the presence of both mitogens, human recombinant EGF and basic FGF. After 7 days of incubation, neurospheres that measured between 50 μm and 100 μm in diameter were counted under an inverted microscope. Clonal efficiency was determined as the ratio between the total cell seeded and the number of neurospheres obtained. An example of this analysis and of stable clonogenic ability in serially subcultured hNSCs is shown in Figure 2B.

#### Differentiation test

A critical feature of hNSC is their multipotency, i.e. their ability to give rise to the three major neural lineages [8]. To show that hNSCs retained stable differentiation capacity throughout culturing, their multipotency was determined by *in vitro* differentiation tests, in which the simultaneous presence of neurons, astrocytes and oligodendrocytes was detected by immunocytochemical labeling (β-tubulin III, neuron marker; GFAP, astroglial marker; GalC, oligodendroglial marker). Neurospheres were mechanically dissociated and the cells were resuspended in the same culture medium used during their routine culturing but in the absence of EGF and in the presence of the sole bFGF. Cells were seeded on a cultrex layer (Cultrex® Basement Membrane Extract, Trevigen) and incubated at 5% O2, 5% CO_2_ and 37°C for 3 days. Then culture media was replaced with DMEM/F12 supplemented with 2% FBS, w/o growth factors [27]. Cells were incubated for an additional 7 days, after which they were fixed with 4% paraformaldheyde and immunostained using a standardized method [8]. An example of such a differentiation test is shown in Figure 2C.

#### Growth factor dependence

Neural stem cells appear to be quite resilient to transformation. Both mouse and human cells depend on the presence of mitogens to undergo proliferation and promptly differentiate upon their removal from the culture [28]. Thus, ensuing growth factor independence is considered as a sign of potential hNSCs transformation. Hence, we routinely monitored growth factor dependence in our GMP-grade hNSCs, in order to provide indirect evidence to their lack of transformation and tumorigenicity. This was evaluated by shifting expanding cells to a culture media without EGF and bFGF and analyzing their growth curve upon further sub-culturing. The latter ought to display a negative slope, confirming the cells’ inability to sustain self-renewal in the absence of mitogenic stimulation, as shown in Figure 2D.

#### Karyotype

**Figure 3:**
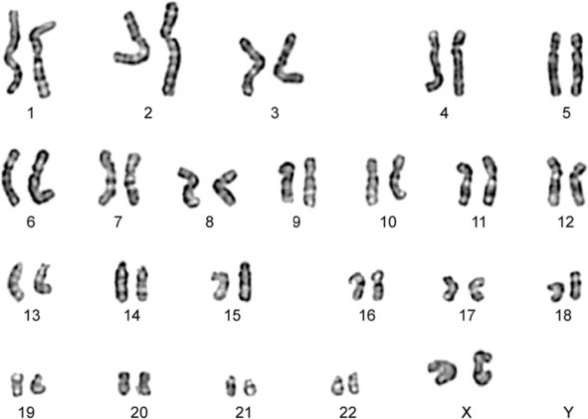
**The cell lines used in this study were confirmed to retain a normal karyotype all throughout passaging.** The figure shows the example of a karyogram performed on the hNSC line from a female donor (46, XX) after seventeen passages. Chromosome G-banding was routinely performed on both the Intermediate Product and the Finished Product. In addition we also tested for karyotype stability the cells that were left in the needle, post-transplantation.

We also monitored the stability of the hNSCs karyotype all throughout passaging in culture. Chromosome G-banding confirmed that the two cell lines used in this study retained their normal, 46 XX or 46 XY karyotype profile all throughout culturing (Figure 3). Forty eight to 120 hours from seeding, cell cultures (higher than 1,000,000 cells) were treated for three hours with colcemid solution (KaryoMAX® Colcemid® Solution, 10 μg/ml) at a final concentration of 0.05 μg/ml to arrest cells in metaphase. Cells were then treated with hypotonic solution (0.075 M potassium chloride solution, SIGMA) and with a fixative solution, prior to proceeding with the karyotype analysis.

#### Tumorigenicity test in vivo

**Figure 4:**
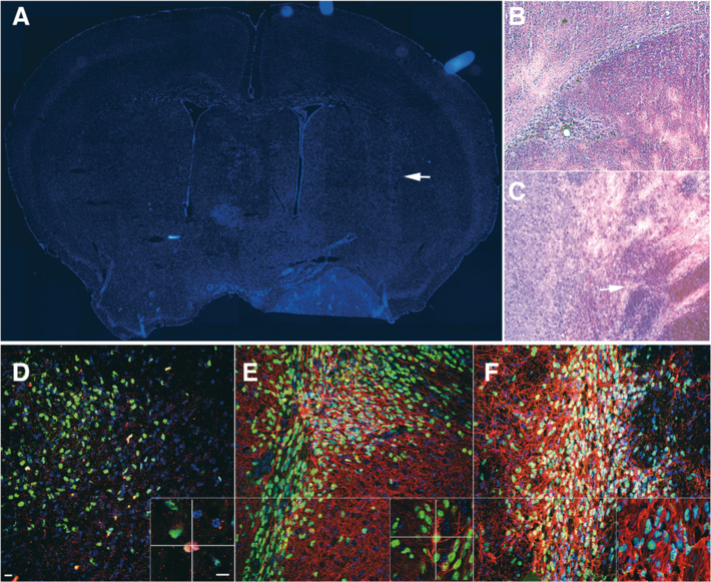
**hNSCs transplant into Nude Mice CNS.** The lateral striatum of nude mice was the target area (**A**, arrow) for the transplantation of normal hNSCs **(B)** or glioblastoma cancer stem cells (GBM; positive graft controls; **C)**. Mice were sacrificed six **(B)** and two months **(C)** after transplantation, respectively. The hematoxilyn/eosyn stain showed that structural organization of the transplanted regions was well preserved in mice tranplanted with hNSCs **(B)**, whereas hypercellularity and anomalous growth and necrosis ensued in regions receiving GBM cells **(C)**. Confocal microscopy of anti-human nuclei staining (huN, green, **D**) showed that hNSCs engrafted efficiently, with only a few human cells retaining residual proliferation activity as shown by co-labeling with the proliferation marker ki67 (red). hNSCs labeled with huN (**E**, green) differentiate into βTubIII+ neurons (**E**, red) and GFAP+ astrocytes (**F**, red). Nuclei are shown by DAPI staining. Scale bars: D**-**E = 15 μm; all insets: 10 μm, bar in inset D.

The lack of tumorigenicity in hNSCs was also evaluated directly by *in vivo* safety testing. Immunodeficient, athymic nude mice, were injected into the lateral striatum (Figure 4A) with 3 × 10^5^ hNSCs. They were sacrificed six months later. Immunohistochemistry analysis showed that, as seen previously [8], hNSCs engraft efficiently, with only a few sporadic cells remaining positive for the human proliferation antigen Ki67. Engrafted cells differentiated into neuronal cells expressing the βTubulinIII antigen and into astrocytes expressing the glial fibrillary acidic protein (GFAP) (Figure 4D, E, F). We observed neither signs of hyperproliferation (only an average of 4.83 ± 1.12% of Ki67 positive cells per transplant were found in the 20 animals analyzed per each cell line (n = 15)) nor tumor formation (Figure 4B). We also ran positive controls to verify that the system used here was truly capable to detect the presence of engrafted cells with tumorigenic ability (positive controls). To this end, we set up a parallel group of transplanted nude mice that received between 50 and 50.000 transformed human neural cancer stem cells [29]. In these animals, immunohistochemistry analysis promptly revealed formation of hypercellular, invasive tumor cell masses as early as 3 months after transplant at the highest concentration and approximately 5 months for the lowest (Figure 4C).

### Surgical procedure

The details of the surgical apparatus and transplantation protocol have been published previously [18]. Briefly, a platform was fixed to the spine through percutaneous posts. After laminectomy (T8-T11) and dural opening a “floating retracting cannula” design was used to place a needle precisely into the ventral horn, using a rigid conformation. After placement, the cannula was retracted and converted to its flexible form to allow it to float jointly liable with the spinal cord. Unlike the previous trial we decided to use a cannula with a broader, 25 gauge diameter (instead of 30) to facilitate cells loading and to minimize their reflux along the needle track during infusion, also in consideration of the high cell concentration of our cell suspension, which was 5 folds higher than those previously used [30]. On average, the time required for the entire surgery procedure was approximately 4 hours. Before implantation cells were counted and suspended in HBSS buffer (Hank’s BSS 1X, PAA) at a concentration of 50,000 cells/μl. After batch release, cells were maintained at 2-8°C for not more than 1 hour prior to implantation. Patients received either unilateral (Group A1) or bilateral (Group A2) NSCs microinjections (3 microinjections on each side) into the lumbar spinal cord. Each microinjection consisted of 15 μl of the above 50,000cells/μl suspension, yielding a total of 750,000 cells per injection site. Standard Monitoring techniques were applied during surgery and included lower extremity somatosensory and motor evoked potentials, when readings proved reliable. All surgical procedures were performed under general anaesthesia using previously described procedures [31]. Cefazolin 1 g IV was administered at the time of dural opening and immediately after surgery.

### Immunosuppression

Methyprednisolone 125 mg IV was administered preoperatively at 2 hours before incision. Patients subsequently received oral prednisone, with a 28-day taper and a dose change each week, declining from 60 mg to 40 mg, 20 mg, and 10 mg orally, every day.

Tacrolimus was administered orally at 0.1 mg/kg twice a day, beginning on post-operative day 1. The drug was titrated so as to maintain blood levels comprised between 5–10 ng/ml for 6 months, after which the treatment was terminated.

### Postoperative care and rehabilitation program

After surgery patients were admitted to the Intensive Care Unit and mobilized. On postoperative day 3 patients were admitted in the neurosurgical unit and on day 5 or 7 (depending on the patient conditions) were transitioned to physical rehabilitation department for a 20-day period of intensive physical therapy. Neurological status, including assessment of muscle strength by MRC and spasticity by Ashworth scale and general examination (including blood pressure), biochemistry (renal function, liver function, glucose, tacrolimus levels) and haematological assessments (full blood and CD4 count) were performed daily during the first week post-surgery. This procedure was carried out weekly for the first three weeks and monthly, thereafter. FVC was performed monthly.

### Discontinuation of the study

The study could be halted at any time either by the Safety Monitoring Board, by the ISS, by the investigator(s) or by the ethics committees, based on any medical event experienced by the patient, which was deemed to be of clinical significance and that might occur after the treatment. Severity was graded according to the modified WHO criteria.

## Results and discussion

### Patients

We received a total of 227 applications to participate in this study, 185 of which turned out to be ineligible since they did not meet the inclusion criteria. A total of 18 eligible patients were summoned for the initial medical screening at the recruitment centres. After the visit and detailed discussion of the informed consent 9 patients withdrew their request to participate. Three of the patients that were enrolled developed respiratory failure during the three months of observation of the disease natural history prior to transplantation. This made it impossible for them to safely undergo surgery. The final cohort of patients included 6 sporadic ALS patients (2 females and 4 males). The principal characteristics of the recruited patients are described in Table 2.

**Table 2:** **Clinical characteristics of patients**

**Patient (Code)**	**Group**	**Age**	**Sex**	**ALS-FRS-R Score**	**FVC**	**Disease duration at entry**	**Follow up duration**
					***(%)***	***(Months from symptoms onset)***	***(Months from surgery to death (†) or to the last visit)***
740	A1	30	M	25	74	60	18
746	A1	57	M	28	64	68	8†
753	A1	54	F	29	83	16	8†
766	A2	35	M	30	82	72	12
767	A2	67	F	35	88	36	8†
779	A2	38	M	24	82	24	7

A1; unilateral hNSCs microinjections.

A2; bilateral hNSCs microinjections.

†; death.

Their median age was 46 years (Range: 30–67). The median duration of the disease from the onset of symptoms to recruitment was 48 months (Range: 16–72). The median ALS-FRS-R score at the study entry was 28 (Range: 24–35), while the median FVC was 82% (Range: 64–83).

### Adverse events

The treatment caused no severe adverse events. All patients were extubated without problems in the operating room and showed no immediate, post-operative respiratory difficulties. The most common adverse event was post-surgical pain, as reported immediately after surgery. This was confined to the injection sites and to the corresponding dermatomes, with only one patient (number 753) experiencing transient, painful spasms in the lower limbs. Pain was mild (WHO II-III) and well compensated with narcotic and non-narcotic analgesics, disappearing an average 4 days after surgery (Range: 1–6). There was no correlation with the number of the injection sites. One patient (number 779) developed deep vein thrombosis in the leg 3 months after surgery, which required anticoagulant treatment. Subsequently a hematoma at the site of surgical scar was observed. MRI confirmed that the blood and oedema at this site were superficial and confined to the epidermal layer. The hematoma showed no clinical neurological correlate and readily resolved upon drainage.

No patients suffered side effects from immunosuppressive treatment. Tacrolimus was well tolerated and did not have to be suspended prematurely. All patients showed tacrolimus blood levels that were within the therapeutic target range, confirming compliance. Renal and liver function, blood and CD3^+^; CD3^+^/CD8^+^ CD4^+^/8^+^ counts remained within normal range.

SEPs and MEPs showed no changes of the sensory and motor conduction time during and following surgery. Bladder ultrasound showed no abnormal post-void residual volumes.

## MRI results

**Figure 5:**
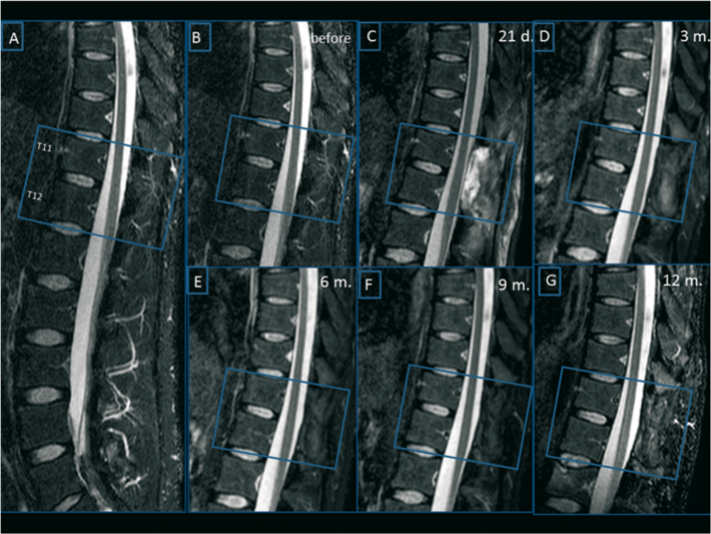
**MRI Follow-up.** T2 weighted sequences acquired on sagittal plane before surgery (images **A**-**B**) and respectively 21 days (image **C**), 3 (image **D**), 6 (image **E**), 9 (image **F**) and 12 months (image **G**) after transplantation. Post-surgical MR scans revealed an expected extradural fluid collection at the site of surgery, which resolved spontaneously. No structural changes were detected after hNSCs transplantation relative to the baseline.

There were no post-procedural complications. In all patients post-surgical MR scans revealed an expected extradural fluid collection at the site of surgery, which resolved spontaneously after 3 to 6 months (Figure 5A-G). Serial MRI showed no structural changes (including tumor or syrinx formation) within the brain and the spinal cord after transplantation relative to the baseline (Figure 5). A 12 months follow-up analysis in 2 out of 6 patients portrayed no significant, long lasting changes in the Apparent Diffusion Coefficient (ADC) and Fractional Anisotropy (FA). At post-surgery day 21 a slight reduction of the FA values was observed, followed by a subsequent recovery at later follow-up scans. ADC, which probes the interstitial space restriction in the case of ischemia or cytotoxic oedema or, conversely, facilitation of water diffusion in the case of increased extracellular interstitial water, showed no evidence of post-transplant damage. Neither brain morphological and signal alterations nor pathological enhancements were detected 12 months after surgery.

### Post-Transplantation hNSCs safety/identity tests

**Figure 6:**
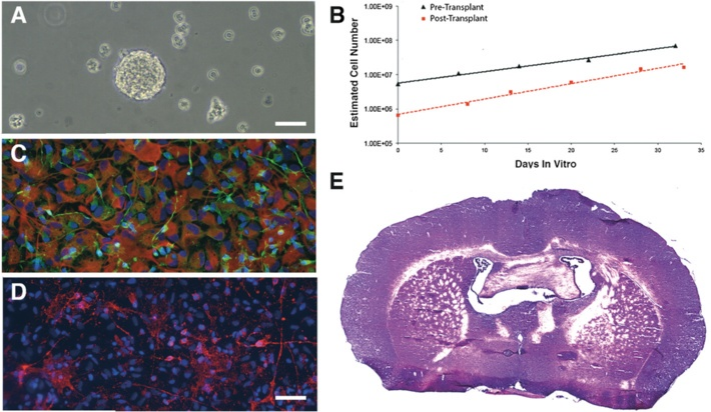
**Post Transplant hNSCs Test. (A)** an example of human neural stem cells that were leftover from the transplant and put back in culture were they re-established typical neurosphere, expanding lines, with a growth profile that mirrored that of the very same cultures prior to the transplant, as shown in **B**. These cells differentiated into neurons (βIII-Tubulin, green, **C**) and astrocytes (GFAP, Red, **C**) and oligodendrocytes (GalC, Red, **D**). **E**: an example of whole brain reconstruction from one out of 10 nude mice that were transplanted into the right lateral striatum with the cells recovered from the transplant and recultured, showing no hyperplastic areas or tumor formation. Bars: A, 100 μm C,D 50 μm, Bar in D.

The few microliters of residual hNSCs suspension that were left inside the injection cannula were harvested at the end of the whole transplantation procedure and put back into culture. By applying the same set of analysis adopted for their initial GMP certification, we were able to determine that the implanted hNSCs had retained the full complement of stem cell properties, including self-renewal and multipotency and lack of tumorigenicity upon in vivo implantation into immunodeficient mice (Figure 6).

An aliquot of the very same cells that were injected into the patients’s spinal cord were fixed the day of the intervention and tested for karyotype stability, as described into methods section. All of the cells used in this study showed a normal chromosomal asset (see example in Figure 3).

### Follow-up

**Figure 7:**
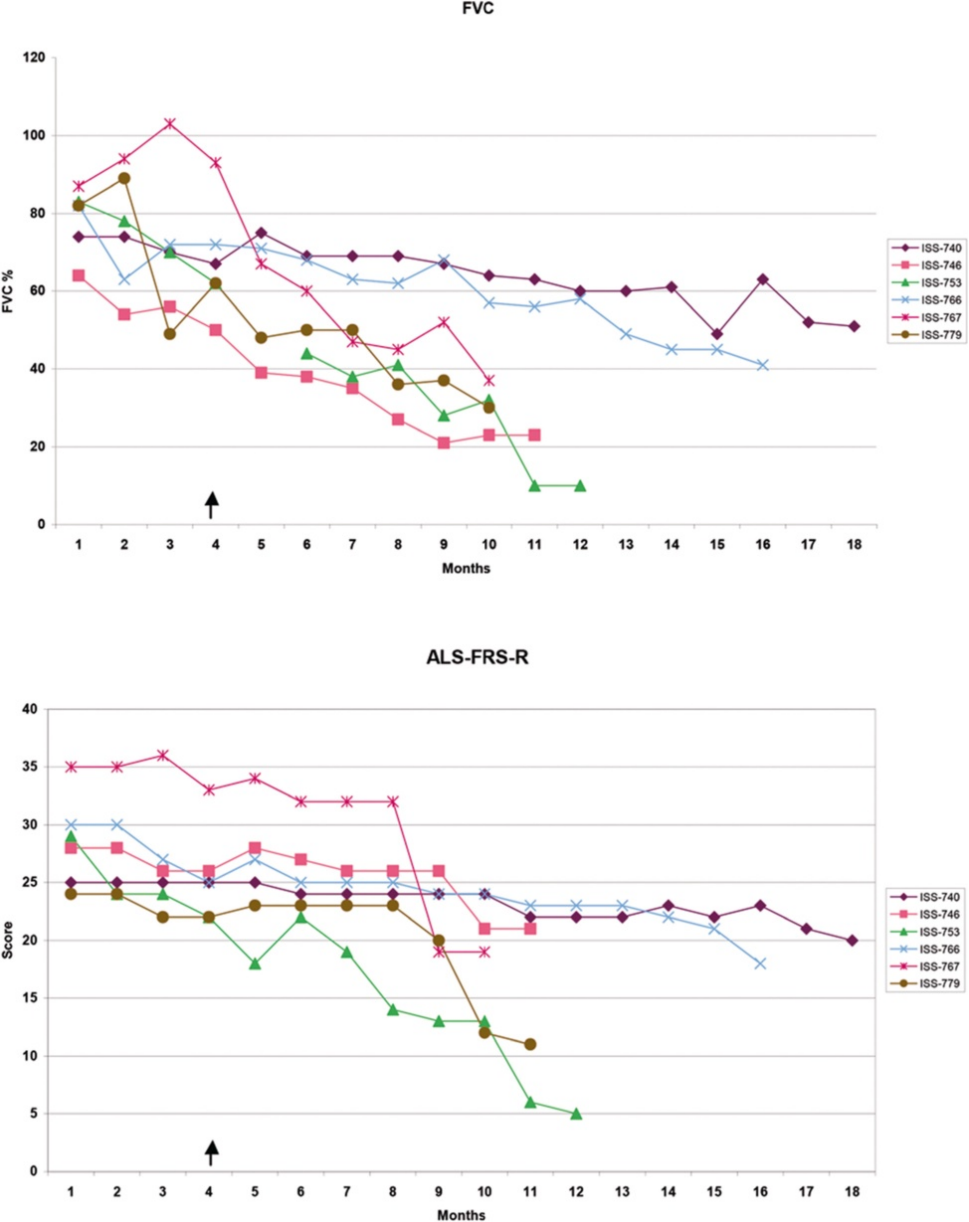
**Clinical follow-up.** Changes of the Forced Vital Capacity (upper panel) and of the ALS-FRS score (lower panel) in the 3-month period of natural history observation and after transplantation. The arrow indicates the time of NSCs transplantation.

Clinical assessments ranging from 6 to 18 months after transplantation showed no acceleration in the course of progression of the disease due to the treatment. No significant modifications in the decline of all clinical and instrumental measures were observed between the pre- and post-transplantation phases (Figure 7).

Patients 740 and 779 showed a transitory improvement in the ambulation sub-score on the ALS-FRS-R scale (from 1 to 2), which persisted 3 and 1 month after surgery, respectively. Patient 753 showed an improvement of the MRC score for tibialis anterior (from 1 to 2) that persisted for up to 7 months after surgery.

**Figure 8:**
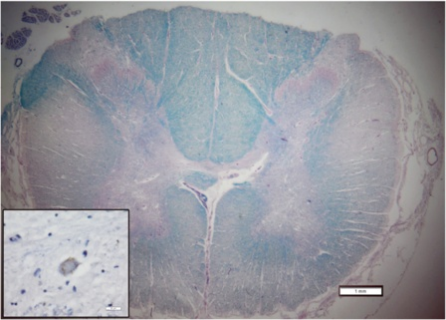
**Representative cross section of the spinal cord in the region of transplantation stained with luxol fast blue and periodic acid Schiff.** There is no apparent disruption of tissue due to injection. Note the degeneration of the cortico-spinal tracts (“lateral sclerosis”). The inset demonstrates a phosphorylated TDP43 inclusion in a remaining motor neuron. Scale bars are 1 mm for the low power and 20 microns for the inset.

Eight months after surgery, patient 753, together with two additional patients (746 and 767), died due to the progression of respiratory failure related to the natural course of the disease. All the three of them had refused PEG and invasive ventilation. The autopsies of patients 746 and 753 documented no other causes of death. The autopsy of patient 767 was not carried out because the relatives declined the necessary consent. Histopathological, post-mortem analysis from the one patient from whom the spinal cord tissue could be harvested showed neither tissue abnormalities nor the presence of hyper-cellular regions (Figure 8).

### Neuropsychological assessment

No negative reactions on the patients’ mood and quality of life were observed. Only patient 753 experienced alterations in his mood, accompanied by depressive symptoms in the post-surgery period. This emerged as a reaction to the paucity of assistance that she/he received, from her/his family and the progressive deterioration of the relationship with her/his spouse. The mood improved after the activation of the domiciliary assistance from the public health services. All patients were interviewed at the last visit and confirmed that they were satisfied by their participation in this study.

This study on six patients with ALS shows that transplantation of hNSCs into the anterior horns of the lumbar spinal cord appears to be a safe procedure that causes no major, short- or medium-term, deleterious effects. Our data are consistent with those reported previously [18,19,32]. Of importance, our findings emerge from a similar experimental and clinical protocol that is comparable to these studies but we, in turn, employed a much higher cell dosage and a milder immunosuppression regimen.

The first result is to be found in the fact that the surgery underpinning intraspinal cell injection in humans appears to be a safe, reproducible and reliable procedure. Also, perspective improvements were made here, particularly concerning the number of implanted cells, that in our study was four and a half times higher than in previous approaches – a maximun of 4.5 × 10^6^ cells/patient used here versus the previous 1 × 10^6^ cells [32]. In addition, we adopted a floating cannula with a larger diameter. This further reduced the risk of the cells clogging the needle, and minimized their flowing back along the walls of the needle track through the cell infusion procedure, irrespective of their higher concentration in our cell suspension.

Second, MRI scans revealed no structural changes or tumor formation in the spinal cord parenchyma all throughout the follow-up period. The analysis of Apparent Diffusion Coefficient (ADC) and Fractional Anisotropy (FA) did not show any damage to the spinal tissue. Of note, FA is a parameter that probes the axonal density. Thus a lesion of the spinal cord should induce a definite and long lasting reduction of FA. In our 18 month-long follow up we observed no significant, long-lasting changes in the FA values. This was further confirmed by the histopathological, post-mortem analysis.

At present, any interpretation of the slight clinical improvement observed in three patients remains highly speculative. Notwithstanding, by taking the evidence available from the literature into account, some cautious hypothesis can still be put forth, if only tentatively. Such effects could be related to the well known ability of NSCs to ameliorate the pathophysiological and neurological traits in many animal models of neurological disorders, including ALS [13]. This may occur through the release of neurotrophic and growth factors, cytokines and immunomodulatory molecules [33] that diffuse into the pathological tissue, thereby eliciting multifaceted, anti-inflammatory and anti-gliosis effects. In agreement with this view, we have recently found that the same cell used in this trial can effectively reduce reactive microglia in the spinal cord of SOD1G93A rats (Vescovi et al., manuscript in preparation). Intriguingly, compelling evidences now exist that implanted NSCs, which integrate and survive predominantly as astroglial cells in the brain parenchyma, exert their immunomodulation ability through secretion of extracellular membrane vesicles (EMVs), influencing the microenvironment through the traffic of bioactive molecules [34,35]. If the above were the case, it would also help to explain as to why these mild clinical improvements may occur irrespective of the total number of injections, as observed here.

An additional, significant finding from this study is the lack of acute or delayed toxicity that might have ensued from the transplantation of our hNSCs lines. The latter are routinely generated anew and banked as tissue becomes continuously available over time. This approach has now allowed us to establish a GMP-certified, clinical grade bank of hNSCs, which is constantly expanding both in size and donors repertoire. Each single hNSCs line is steadily and plentifully expandable *ex vivo*, with multipotentiality and functional characteristics remaining stable and certified throughout time. Thus, not only does this approach provide a broad array of different hNSCs lines for future studies – allowing for determining if cells from different regions or developmental ages may be more amenable for cell therapy in different pathological contexts – but also enables us to carry out this whole phase I trial using cells from a maximum of two donors.

Previous pre-clinical data from our lab using these same hNSCs pointed to a low expression profile of HLA molecules. Furthermore, negligible immunological reaction was observed following intracerebral transplantation, upon transient (peri-transplantation) immunosuppression [10]. Hence, in considering that ALS patients at advanced stages of the disease might be weaker and more vulnerable to pharmacological toxicity, in the study reported here we adopted a reduced immunosuppression regimen as compared to that employed by Glass and colleagues [19]. As expected, we observed a smaller cohort of side effects arising from the immunosuppressive treatment.

While transplantation of cells from human foetuses is an accepted technique, it is undeniable that such an approach is often met with ethical and moral concern, particularly outside the scientific community. At times, heated public debates have escalated to worrisome levels due to the fact that, to date, this tissue has always been derived from therapeutic or procured abortions. This also explains as to why approval from ethical committees may encounter significant hurdles when the use of foetal material is involved. To the best of our knowledge, this is the first time that a procedure for the isolation of hNSCs is successfully carried out from foetuses that were certifiably deceased *in utero* due to natural causes. Also, tissue procurement in this study took place according to the same international guidelines that are in place for the donation of organs for therapeutic transplantation. Hence, this provides an approach to hNSCs transplantation that is free from the ethical concerns that may arise from the use of foetal human tissues.

In all experimental and clinical transplantation studies on hNSCs, the lack of univocal hNSC markers makes it difficult to precisely define the actual composition of the transplanted cell suspension. This matter is further compounded by the fact that implanted cells undergo significant stress due to their preparation for transplantation, their transportation and loading into the needle and throughout the injection process. Up until now, it could not be ruled out that the cells death that inevitably occurs throughout these procedures might significantly or even prevalently affect the hNSCs pool in the injected cell suspension. As paradoxical as it may seem, the chances still exist that the stem cell content in the cell suspension that eventually reaches the patient’s own tissue in hNSCs transplantation procedures may drop significantly, even to the point that the final graft might actually contain negligible amounts of *bona fide* brain stem cells. Here we were able to recover the few cells that were left in the injection needle following transplantation and to examine them thoroughly. By this, we were able to show that the cells that were injected into the ventral horns of the spinal cord of ALS patients were, in fact, the same hNSCs suspension that were released from the cell factory that, as such, did retain key features of stemness and lack of tumorigenicity (Figure 6).

We are now routinely carrying this procedure out. This introduces an additional level of control on the cell composition and quality that should help improve standardization of hNSC-based cell therapies in future trials.

We have been able to reproducibly and quite extensively expand hNSCs *ex-vivo*. Thus, this whole trial is actually based on the use of only two donors. The use of such a limited number of donors inherently reduces the inter-treatment variability that may arise from the implantation of different hNSC lines into different patients.

Very few studies in the scientific literature report the results of clinical trials with stem cell transplantation. These results are not definitive and no trial has been replicated in multiple centres. Experimental neural transplantation inevitably involves only relatively small groups of patients, with a corresponding loss of statistical prowess. Hence, only international collaboration may accomplish the most effective progress in stem cell clinical trial. This is the first report of an international coordinated effort about the cell therapy and transplantation approach in ALS patients. By utilizing a methodology similar to that adopted by our collaborators [18], we have reproduced the safety of the approach and provided an improved ability to compare the relative efficacy of the different cell types, also factoring out variance in the approach to delivery. A large multidisciplinary team of cell biologists, neurologists and neurosurgeons with significant experience in experimental cell therapy and in the treatment of ALS are involved in our studies. These researchers worked closely with regulatory agencies (AIFA, ISS), patient advocacy groups and ethical regulatory bodies. Moreover this is the first clinical trial with hNSCs that is run entirely by a not-for-profit organization, strictly on a charitable fund raising basis.

We are now broadening the import of this trial by testing intraspinal injections into the cervical spinal cord (C3-C4 level) of 12 ambulatory patients.

## Conclusions

We can conclude that, while these results are not definitive, it appears that transplantation of human fetal neural cells in the lumbar spinal cord is a safe procedure. This technique has now been freed of any ethical concerns arising from the use of human fetal tissue. In addition, up to five fold more cells can now be safely implanted into the human spinal cord and a milder immunosuppression regimen adopted in ALS patients in the absence of related adverse effects.

We will next compare our results with the findings from the recently concluded trial at Emory University, which will help both groups to extend and deepen their conclusion on the use of cell therapy in ALS.

The results from this investigation also describe a cell therapy platform that will allow broadening the number and reproducibility of cell therapy clinical trials for ALS and, other neurological disorders. These can now be carried out under more standardized conditions and will be based on a more homogenous repertoire of clinical grade, donor hNSCs, which also avoid ethical concerns.

## Abbreviations

ALS: Amyotrophic lateral sclerosis
hNSC: Human neural stem cell
GMP: Good manufacturing practice
ALS-FRS-R: Amyotrophic lateral sclerosis-functional rating scale-revised
MRC: Medical Research Council
PEG: Percutaneous Endoscopic Gastrostomy
MN: Motoneuron
MSC: Mesenchymal stem cell
NPC: Neural progenitor cell
FDA: Food and Drug Administration
ISS: Istituto Superiore di Sanità
AIFA: Agenzia Italiana del Farmaco
EudraCT: European Clinical Trials Database
FVC: Forced vital capacity
POMS: Profile of mood state
SEIQoL-DW: Schedule evaluation of individual quality of life direct weighting
SC: Spinal cord
MRI: Magnetic resonace imaging
DTI: Diffusion tensor imaging
FA: Fractional anisotropy
ADC: Apparet diffusion coefficient
ROI: Region of interest
MPR: Multi planar reformat
EMA: European Medicine Agency
DMSO: Dimethyl sulfoxide
PBS: Phosphate buffered saline
FBS: Foetal bovine serum
IPC: In process control
LAL: Limulus amebocyte lysate
DMEM: Dulbecco minimal essential medium
EGF: Epidermal growth factor
bFGF: Basic fibroblast growth factor
GFAP: Glial fibrillar acidic protein
GalC: GalactosylCeramidase
HBSS: Hank’s balanced salt solution
WHO: World Health Organization
SEP: Sensitive evoked potential
MEP: Motor evoked potential
ADC: Apparent diffusion coefficient
HLA: Human leukocyte antigen
